# Temporal Dynamics of Nectar and Pollen Production in Protandrous Flowers of *Nigella damascena*

**DOI:** 10.3390/plants15060928

**Published:** 2026-03-17

**Authors:** Zuzanna Łabęcka, Bożena Denisow, Monika Strzałkowska-Abramek

**Affiliations:** Department of Botany and Plant Physiology, University of Life Sciences in Lublin, 15 Akademicka St., 20-950 Lublin, Poland; zuzannalab@gmail.com (Z.Ł.); monika.strzalkowska@up.edu.pl (M.S.-A.)

**Keywords:** protandry, nectar production, nectar sugars, pollen production, honey bees

## Abstract

This study examined nectar and pollen production as well as pollinator visitation in *Nigella damascena* (Ranunculaceae), an annual ornamental and seed crop, over two flowering seasons. Flower anthesis lasted 6–7 days, with protandry: the male phase began on the first day, and pollen presentation continued until corolla senescence. Peak stigma receptivity occurred in 5-day-old flowers, resulting in a partial overlap of male and female functions between days 5 and 7. Nectar was secreted by petal-derived structures, with secretion beginning in 1-day-old flowers and steadily increasing, peaking on the day of maximum stigma receptivity. The nectar sugar composition differed between floral phases; it was sucrose-dominant in the male phase and sucrose-rich in the female phase. Significant year effects were observed for flowering abundance, nectar traits (volume, sugar production, concentration), and pollen output. Flowers were visited predominantly by honey bees, but bumblebees, solitary bees, and dipterans were also recorded. These results demonstrate that floral reward traits vary between years and contribute to differences in the temporal availability of nectar and pollen resources.

## 1. Introduction

In animal-pollinated angiosperms, nectar and pollen are the main floral rewards, serving to attract pollinators and ensure reproductive success [1,2,3]. Among these, nectar is a carbohydrate-rich secretion that provides essential energy to foraging pollinators, thereby mediating plant-pollinator interactions and influencing pollination efficiency [4,5]. Pollen provides essential proteins, lipids, macro- and microelements, vitamins, and hormones critical for pollinator nutrition [6,7].

Within the Ranunculaceae family, floral rewards vary among species, with some flowers offering both nectar and pollen, while others provide pollen alone [8]. When present, nectar is typically secreted by specialized structures called nectaries [9]. These nectaries exhibit considerable morphological diversity and may originate from various floral organs, including modified petals (commonly referred to as “honey leaves”), staminodes (sterile stamens), or components of the gynoecium [10,11,12,13]. The quantity, temporal dynamics, and chemical composition of nectar, including the sugar concentration and the ratios of glucose, fructose, and sucrose, are highly variable, both interspecifically and temporally, often fluctuating across different phenological stages of the same flower [14,15,16,17]. Variation in nectar traits is known to affect pollinator attraction and can interact with floral morphology and reproductive strategy to shape pollination success [18,19].

There are 18 taxa classified under the genus *Nigella* (Ranunculaceae) [20], mostly native to the Mediterranean region, with a range extending from West Asia to northern India [21]. Most of the taxa occur in the wild; however, some are also cultivated in agricultural fields, primarily for culinary and medicinal purposes, owing to their well-recognized phytochemical composition and pharmacological effects [22,23,24]. In Middle Eastern countries, species such as *N. sativa* also serve as important nectar sources: commercial honey is produced from black cumin, highlighting the dual agricultural and apicultural significance of the species [25,26,27]. Among *Nigella* representatives, *N. damascena* is a relatively underexplored species. It is primarily cultivated as an ornamental plant, with its cultivation having increased significantly in recent years [28].

The objective of this study was to investigate the flowering phenology of *Nigella damascena*, including the onset and duration of flowering, as well as patterns of nectar and pollen production, total nectar sugar and pollen output per unit area, and attractiveness to foraging insect groups. The observations were conducted on an introduced population grown outside its native range, in experimental plots in a botanical garden in Poland. Preliminary observations indicated that the flowers are protandrous, prompting a detailed examination of nectar dynamics and sugar composition across the male and female floral phases. Unlike previous studies on *Nigella* species, this work integrates sexual phase dynamics with quantitative analyses of nectar and pollen production, nectar sugar composition, and pollinator visitation across two flowering seasons.

## 2. Results

**Figure 1:**
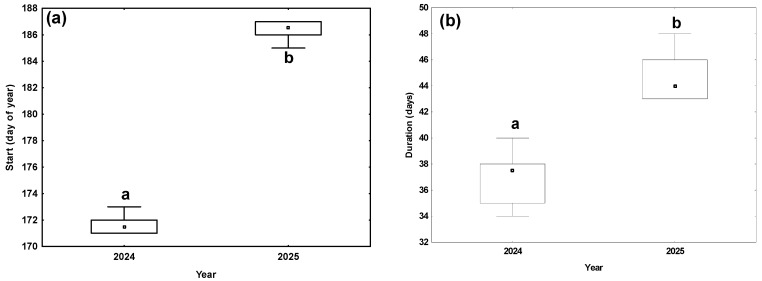
Variation in the flowering phenology of *N. damascena* in 2024 and 2025: (**a**) start of flowering (day of year); (**b**) duration of flowering (days). The boxes represent the interquartile range; the dots in the boxes indicate the median values, and the whiskers show the minimum and maximum values. Significant differences between years (*p* < 0.05) were verified using the Mann–Whitney U test and are marked with lowercase letters.

The flowering phenology of *Nigella damascena* exhibited significant interannual variation between 2024 and 2025. In 2024, flowering commenced earlier, and the overall flowering period was significantly shorter compared to 2025 (Mann–Whitney U test; for flowering onset: Z = −2.85, *p* < 0.001; for flowering duration: Z = −2.81, *p* = 0.004; Figure 1).

**Figure 2:**
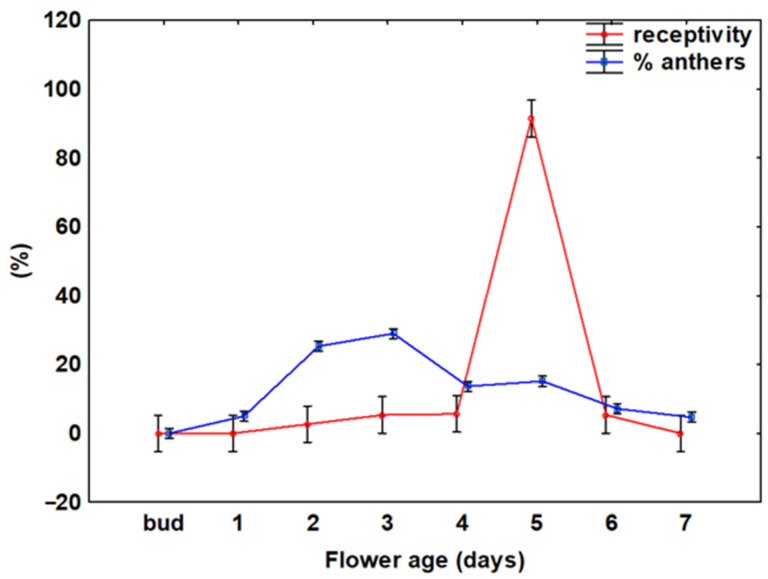
Stigma receptivity (red line) and anther dehiscence (blue line) relative to the flower’s life –span. Values represent the percentage of flowers showing anther dehiscence or stigma receptivity. Vertical bars represent 0.95 confidence intervals.

The number of flowers produced per individual plant and per unit area differed between the two years (Table 1). The average life span of an individual flower was 6.6 ± 1.0 days (mean ± SD). The flowers are protandrous, with the male phase beginning on the first day of anthesis. The male phase was easily distinguished by characteristic stamen movements. Initially, the stamens are positioned upright in symmetrically arranged clusters surrounding the ovary. Following anthesis, the filaments of the outermost stamens begin to elongate and bend down slightly, assuming an almost horizontal orientation. Anther dehiscence was observed during the entire anthesis, with the male phase noted in 1–4 day-old flowers (Figure 2). Most anthers dehisced in 2- and 3-day-old flowers. From the 5th day of the flower’s life span, the overlapping of male and female phases was observed.

Stigma receptivity was absent during the early floral stages (1–3 days of anthesis), peaked on the 5th day of anthesis, with 85% of flowers exhibiting receptive stigmas, and declined significantly in older flowers (6–7 days of anthesis). A similar pattern was observed in both study years.

In *N. damascena*, nectar was produced by specialized nectaries, i.e., petal-derived structures situated within a corolla formed by five sepals. Nectar was produced and accumulated at the base of the petal tube that forms a chamber measuring approximately 5.17 ± 0.36 mm (mean ± SD) in length. Each flower had 7–9 such nectary structures.

**Figure 3:**
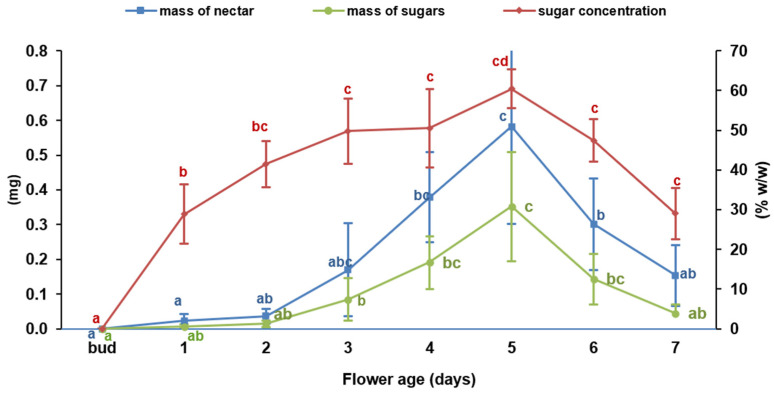
Nectar dynamics during successive days of the flower’s life span. The graph illustrates changes in mean nectar mass (blue line) and mean sugar mass (green line), measured on the primary Y-axis (mg), and mean sugar concentration (red line), measured on the secondary Y-axis (% *w*/*w*). Data points designated by distinct lowercase letters indicate statistically significant differences (*p* ≤ 0.05).

The flower age influenced the amount of secreted nectar, nectar sugar concentration, and sugar content (*p* < 0.0001). Nectar secretion was absent in floral buds and minimal in 1- to 2-day-old flowers (Figure 3). Secretion and sugar content increased in intensity in 3- to 4-day-old flowers, reaching a peak on the 5th day of anthesis. Thereafter, nectar production and sugar content declined in older flowers.

Nectar production in *N. damascena* flowers exhibited significant interannual variation, with approximately 50% higher nectar volumes recorded in 2025 compared to 2024 (year effect: F_1,10_ = 11.46, *p* = 0.006; Table 1). In 2024, the mean nectar volume per flower was 0.58 ± 0.22 mg, increasing to 0.97 ± 0.12 mg in 2025. The sugar concentration also differed significantly between years (F_1,10_ = 7.59, *p* = 0.02), with higher concentrations recorded in 2024 than in 2025. A significant year effect was likewise observed for total sugar content per flower (F_1,10_ = 7.19, *p* = 0.02), with ca. 65% greater sugar content documented in 2025. In the nectar profile, glucose, fructose, and sucrose were detected (Table 2). In addition, trace amounts of maltose were present during the female phase. The composition of the nectar varied between the floral phases. Nectar was sucrose-dominant in the male phase (sucrose 70.7%, glucose 17.4%, fructose 11.9% of total sugars) and sucrose-rich in the female phase (sucrose 49.0%, glucose 36.9%, fructose 14.1%), according to the classification of Baker and Baker [4]. The observed differences in the nectar sugar composition between the floral phases were consistent across samples, indicating a clear phase-related pattern. Differences in the sucrose and hexose ratios between the floral phases were detected.

**Figure 4:**
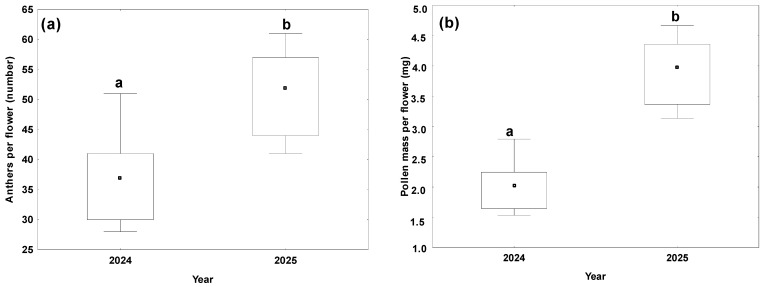
Anthers per flower (number) and pollen mass per flower (mg) observed in 2024 and 2025: (**a**) anthers per flower; (**b**) pollen mass per flower (calculated as the number of anthers multiplied by mean pollen mass per anther). The boxes represent the interquartile range; the dots in the boxes indicate the median values, and the whiskers show the minimum and maximum values. Values with different letters denote statistically significant differences (*p* ≤ 0.05) between the study years.

The androecium, bearing numerous anthers, began to dehisce on the 1st day of anthesis and continued progressively throughout the floral life span. Anther dehiscence was absent in the buds. A significant difference in anther number per flower was observed between years (F_1,18_ = 20.60, *p* = 0.0002), with fewer anthers developed in 2024 compared to 2025 (mean = 36.8 ± 7.1 and mean = 51.2 ± 6.9, respectively; Figure 4).

A significant year effect was observed for pollen mass both at the anther level (F_1,6_ = 20.60, *p* = 0.0002) and at the whole-flower level (F_1,18_ = 81.5, *p* < 0.0001). On average, flowers in 2025 produced nearly twice as much pollen (3.91 ± 0.53 mg) as those in 2024 (2.01 ± 0.39 mg; Figure 4).

**Figure 5:**
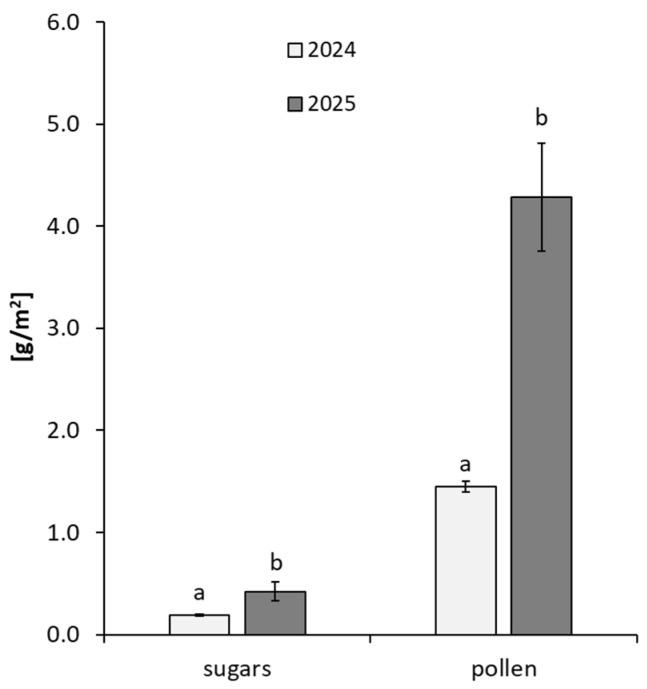
Mean total sugar mass and mean total pollen mass (g/m^2^) in 2024–2025. Vertical lines denote the standard deviation (SD). Significant differences between years (*p* < 0.05) are marked with lowercase letters (a, b).

Across the years, overall sugar production ranged from 0.19 to 0.42 g/m^2^, whereas pollen output ranged from 1.5 to 4.3 g/m^2^ (Figure 5).

**Figure 6:**
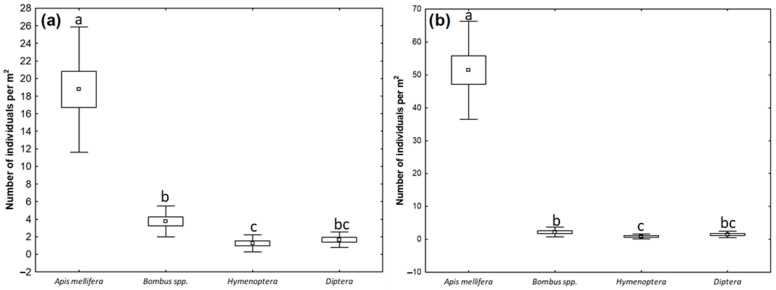
Abundance (number of individuals per m^2^) of insect groups visiting *N. damascena* in 2024 (**a**) and 2025 (**b**). The boxes represent mean ± standard error (SE), the whiskers indicate mean ± standard deviation (SD), and the dots inside the boxes denote mean values. Different lowercase letters indicate statistically significant differences among insect groups within a given year (*p* ≤ 0.05, Kruskal–Wallis test).

The frequency of pollinator visits to *N. damascena* flowers differed significantly among insect groups (Kruskal–Wallis test: H (3, N = 96) = 65.2, *p* ≤ 0.05) (Figure 6). Honeybees were the dominant visitors, with a mean visitation rate of 35.1 ± 20.2 individuals per m^2^. Other insect groups contributed only marginally to overall visitation activity and occurred at similarly low abundances: *Bombus* spp. (3.0 ± 1.7 individuals per m^2^), solitary bees (1.0 ± 0.9 individuals per m^2^), and dipterans (1.6 ± 0.9 individuals per m^2^). Year-to-year differences in abundance were observed for honey bees (Z = 3.98, *p* < 0.001) and bumble bees (Z = 2.04, *p* = 0.040), whereas the distributions of solitary bees and dipterans were similar between the study years (*p* > 0.05) (Figure 6).

## 3. Discussion

The floral biology of *N. damascena* is defined by protandry and well-coordinated temporal patterns of nectar and pollen production. Our results show that nectar secretion and sugar content increase with floral age, peaking around the onset of the female phase, while pollen is available throughout the entire life span.

### 3.1. Flowering Time and Anthesis Characteristics

The flowering of *N. damascena* occurred from mid-June/beginning of July to the end of July/mid-August, which is consistent with previous reports, as optimal sowing typically occurs in April, resulting in flowering from July through late August [29]. This period is particularly important for pollinator support, as it coincides with the “summer food gap” reported for many landscape types across Europe.

The species exhibited a relatively long anthesis period, with individual flowers remaining open and functional for approximately 6–7 days. Comparable duration of anthesis in other *Nigella* species, i.e., *N. sativa*, has been documented by Diwakar et al. [30] and Abu-Hammour & Wittmann [31]. In the species analyzed in our study, the flower functionality is likely a key adaptive trait influencing pollination ecology. The protandrous flowers observed in *N. damascena* have also been reported in other Ranunculaceae species (e.g., [32]), and protandry serves primarily to promote cross-pollination and reduce autogamy. In *N. damascena*, protandry was accompanied by a distinctly short period of stigma receptivity, typically lasting only one day. In most dichogamous species, such brief receptivity is considered an adaptation to reduce within-flower self-pollination [33]. Interestingly, in *N. damascena*, the period of stigma receptivity coincided with maximum nectar secretion, potentially promoting pollinator visitation during the flower’s female phase. A similar combination of functional protandry and overlapping sexual phases has been documented in related Ranunculaceae species [31], suggesting that such a strategy may be common in the genus.

### 3.2. Temporal Dynamics of Nectar Production and Sugar Composition

In *N. damascena*, nectar is produced at the base of tubular structures originating from modified petals. This is consistent with the general pattern reported for the Ranunculaceae, where nectaries frequently occur in petal spurs [10]. Nectar production begins shortly after flower opening, peaks around the fifth day of anthesis, and then declines. Similar age-dependent nectar secretion patterns have been reported in many species, highlighting the influence of flower age on both the quantity and quality of floral rewards (e.g., [32,34,35]). Such temporal dynamics likely reflect physiological constraints and adaptive strategies that maximize pollinator attraction when flowers are most fertile [36]. Given the substantial energetic cost of nectar synthesis, requiring up to 5–37% of a flower’s daily photosynthetic output [37,38,39], such age-dependent changes may optimize the plant’s overall energy economy. Many species can reabsorb uncollected nectar or remaining sugars from nectar [36], suggesting that nectar reabsorption is an effective mechanism for conserving energy and recycling resources [40].

We found interannual variation in nectar production (ca. 50% higher volume in 2025 vs. 2024; higher sugar concentration in 2024). Such variability is consistent with previous findings showing that year-to-year variability in nectar traits (nectar volume and sugar concentration) is attributed to different external factors [19,36,41,42].

The present study demonstrates that the nectar of *N. damascena* exhibits a predominance of sucrose; however, the proportion of sucrose differed between the floral phases. The nectar can be classified as sucrose-dominant in the male phase and sucrose-rich in the female phase, according to the criteria of Baker & Baker [4]. Within the Ranunculaceae, both sucrose-dominant and sucrose-rich nectars have been reported in species with deep floral spurs, such as *Aquilegia*, *Aconitum*, and *Delphinium* [19,43,44]; in taxa possessing tubular nectaries derived from modified petals, for example, *Helleborus* spp. [17,45]; and in open, disc-like flowers such as *Ranunculus* spp. [46].

Typically, sucrose-rich nectars are associated with long-tongued pollinators, such as bees and Lepidoptera, while hexose-dominant nectars tend to be linked to short-tongued or generalist pollinators. However, empirical evidence has shown that this relationship is not always straightforward. Weak or non-significant correlations between nectar traits and pollinator types have been documented in several plant groups, including species of Gesneriaceae [47], multiple families of South American temperate forests [48], and Mediterranean floras [14]. Moreover, large-scale comparative analyses encompassing more than 2000 species suggest that pollinator dietary preferences explain only a fraction of the observed global variation in nectar sugar composition [49]. It therefore appears that the nectar sugar composition in *N. damascena* is influenced not only by pollinator-mediated selection but also by other factors, including phylogenetic constraints, evolutionary history, and biogeographical context.

### 3.3. Pollen Production and Male Reproductive Investment

Male reproductive function in *N. damascena* is characterized by sequential anther dehiscence and substantial pollen production throughout the floral life span. The progressive dehiscence of anthers throughout the floral life span suggests a temporal mechanism that enhances pollen dispersal efficiency in *N. damascena*. Sequential anther opening is a common feature in many entomophilous species and is often interpreted as a strategy to prolong pollen availability and increase the likelihood of pollinator visitation across multiple days [50,51,52]. The absence of anther dehiscence in floral buds indicates that pollen release is strictly restricted to anthesis, which may reduce the risk of self-pollination and promote outcrossing [53].

The significant interannual variation in pollen mass produced per flower indicates that male reproductive investment in *N. damascena* is plastic and responsive to environmental and physiological conditions. In 2025, flowers developed more anthers and yielded nearly twice as much pollen as those in 2024, suggesting that abiotic factors such as temperature, nutrient availability, or water stress may influence anther development and modify the distribution of resources devoted to gametophyte development [54,55]. Year-to-year differences in pollen output have been observed across many temperate herbaceous plants [52,56].

The amount of produced pollen (2.01–3.91 mg per flower) suggests that *N. damascena* can be considered a high pollen-yielding species, producing amounts comparable to or even 2–3 times greater than those reported for other Ranunculaceae members regarded as good pollen producers, e.g., *Ranunculus lanuginosus* (2.9 mg per flower) [57] or *Aquilegia vulgaris* (3.2 mg per flower) [58].

### 3.4. Pollinator Visitation Patterns and Local Context

The composition and abundance of insect visitors to *N. damascena* flowers varied among pollinator groups and between study years. Visitation patterns reflect both floral reward characteristics and the local pollinator assemblage shaped by the landscape context.

In both study years, the floral visitors of *N. damascena* were predominantly honey bees (*Apis mellifera*), consistent with the findings of Zaitoun et al. [25] and Liao et al. [59], who reported a dominance of honey bee activity and the presence of bumblebees on the flowers of this species under semiarid conditions. The dominance of *A. mellifera* likely reflects both floral reward traits and the local pollinator pool, including the presence of managed colonies in the study area. However, discrepancies were observed in the assemblage of other insect visitors; for example, Liao et al. [59] documented the presence of wasps, which were not detected at our study site, while Zaitoun et al. [25] did not report any dipteran species. In fact, insect assemblages and their visitation frequencies to specific plant species are influenced by a complex interplay of factors, including the quantity and chemical composition of floral rewards (nectar and pollen) as well as floral scent profiles. Additionally, the local diversity and population density of pollinators play a critical role in shaping their foraging behavior and visitation patterns [60]. Plant-pollinator interactions are further shaped by biotic and abiotic factors, including the presence of co-flowering species and interspecific competition among pollinator groups [61,62]. In our observations, honey bees were the most frequent floral visitors, which may also reflect their local abundance, as the Botanical Garden where the observations were made hosts an apiary. Year-to-year variability in the abundance of honey bees and bumble bees may reflect differences in environmental conditions, floral resource availability, or colony dynamics influencing forager activity [63].

In summary, *N. damascena* has long-lasting flowers that show distinct protandry, progressive anther dehiscence, age-dependent stigma receptivity, and tightly coordinated patterns of nectar and pollen release. Nectar secretion and sugar content increase with floral age, peaking around the onset of the female phase, accompanied by corresponding shifts in the proportions of individual nectar sugars. The species functions primarily as a pollen, rather than sugar, provider. Importantly, nectar production, sugar content, anther number, and pollen mass exhibited marked interannual variation. These findings highlight the strong influence of environmental conditions on both male and female floral functions and demonstrate that year-to-year fluctuations can significantly alter the quantity and quality of floral resources. Such fluctuations have to be considered when interpreting plant-pollinator interactions under variable environmental regimes. This species may contribute locally to pollinator resource availability, particularly during periods of reduced floral forage.

### 3.5. Study Limitations

The findings of this study should be interpreted in light of several limitations related to spatial, temporal, and methodological constraints. These factors may influence the extent to which the observed patterns can be generalized beyond the study system.

This study was conducted over two consecutive flowering seasons and at a single study site, which limits the extent to which the results can be generalized to other environmental conditions or geographical regions. The interannual variation in nectar and pollen traits observed in this study highlights the importance of longer-term, multi-site investigations to fully capture the range of variability in floral resource production.

In addition, the nectar sugar composition was analyzed based on a limited number of samples and was therefore intended to provide a descriptive and comparative assessment between floral sexual phases rather than population-level inference. Consequently, the phase-related differences in the nectar sugar composition should be interpreted as indicative trends that require confirmation through broader sampling.

Finally, the pollinator visitation patterns may have been influenced by the local landscape context, including the presence of managed honey bee colonies in the study area and the availability of alternative floral resources. Therefore, the observed visitation frequencies should be interpreted in the context of local pollinator assemblages rather than as species-wide preferences.

## 4. Materials and Methods

### 4.1. Study Area and Study Species

The study was conducted during 2024–2025 at the Botanical Garden in Lublin, Poland (51°16′ N, 22°30′ E), situated at an elevation of 200 m a.s.l. The research site lies within the temperate climate zone, classified as Dfb according to the Köppen–Geiger system [64]. The experimental plots we arranged in 6 replications (1 m^2^ each) were established across distinct areas within the Ornamental Plants Section. All plots were located on loess-derived soil (pH = 6–7). In both years, the seeds were sown on 11 April, the optimal time for this species [29].

The study species was *Nigella damascena* L. (Ranunculaceae), an annual flowering herb native to southern Europe. It has spread to northern parts of Europe, where it is not native, and also occurs in North Africa and SW Asia, typically in damp, uncultivated habitats [65]. The flowers are bisexual and radially symmetrical, with showy, petaloid sepals that are typically blue in color [10,66]. The petals, commonly referred to as honey leaves, are much smaller than the sepals and are structurally divided into upper (inner) and lower (outer) lobes [25]. Two glistening pseudonectaries are present on the dorsal surface of each petal; these structures do not secrete nectar but likely act as visual or tactile guides for pollinators [59]. The androecium comprises numerous stamens with longitudinally dehiscent anthers [28]. The gynoecium consists of 2–10 centrally positioned, fused carpels [10].

### 4.2. Flowering Observations—Phenology, Anthesis, and Duration of Sexual Phases

To assess flowering phenology (i.e., onset and duration), we recorded the flowering start date, defined as the day when approximately 10% of flower buds had opened, and the flowering end date, marked by the wilting of around 80% of the flowers [8]. Observations were conducted across six experimental plots.

The flower’s life span and changes in individual flowers were monitored. The floral life span was defined as the temporal interval extending from the flower opening to the completion of pollen release by the final dehiscing anther and wilting of nectaries and petals, representing the period during which floral resources (e.g., pollen and/or nectar) are potentially available to pollinators. Flower development was monitored in randomly selected and labeled flowers of different individuals (in total, n = 24 flowers were monitored per year). The flowers were checked bi-daily at 09:00–10:00 and 16:00–17:00 (EET, Eastern European Time). To record the number of anthers dehisced daily, opened anthers were gently removed with tweezers. We also assessed stigma receptivity through the detection of peroxidase activity (n = 24 flowers per year). To this end, styles were treated with 30% hydrogen peroxide (H_2_O_2_), following the protocol described by Dafni & Maués [67]. The presence of peroxidase activity was indicated by the formation of oxygen bubbles on the stigma surface. Observations were conducted using a Nikon SMZ-2B stereomicroscope.

### 4.3. Floral Reward—Nectar Production

The number of modified nectaries was established, and the length of the nectary chamber was measured (n = 20 nectaries). Nectar was collected using calibrated micropipettes, following the method of Jabłoński [68]. To prevent nectar removal by floral visitors, flowers were enclosed in fine tulle mesh bags (<0.5 mm mesh size). Sampling was conducted between 10:00 and 12:00 EET.

To investigate nectar secretion dynamics and sugar accumulation over floral development, nectar was collected at the bud stage and subsequently on each day of anthesis up to the seventh day of the flower’s life span. To assess nectar production throughout the flower’s life span, flowers were marked at the bud stage (just before opening), and a different subgroup of flowers of known age was sampled on each day of anthesis to infer the nectar production pattern. At each time point, nectar was collected using glass micropipettes. For each time point, we collected n = 6 samples (n = 2 per 3 separate days). Each sample collected at the specific time points consisted of nectar pooled from 15 to 20 individual flowers. Micropipettes containing nectar were reweighed using an analytical balance (RADWAG, Radom, Poland) to determine the nectar mass. The nectar sugar concentration (% *w*/*w*) was measured by placing the extracted nectar onto the prism of a refractometer (PZO, Warszawa, Poland). The sugar mass per flower was subsequently calculated based on the nectar mass and sugar concentration.

To determine the nectar sugar profile, flowers were sampled separately during the male (3rd day of flower age) and female (5th day of flower age) stages, reflecting the protandry in the flowers. Nectar was obtained from multiple individuals (n = 8). The sugar composition was analyzed using a Shimadzu HPLC system (Kyoto, Japan) equipped with a degasser (DGU-14A), binary pump (LC-10AT VP), column oven (CTO-10A VP), diode array detector (SPD-M20A Prominence), and autosampler controller (SCL-10VP). Separation was achieved on a SUPELCOGEL™ column (Sigma-Aldrich/Merck; Darmstadt, Germany) using an acetonitrile:water (80:20, *v*/*v*) mobile phase at a flow rate of 1.5 mL min^−1^. The column and detector were maintained at 30 °C, and the injection volume was 20 µL. Chromatograms were processed using POL-LAB CHROMA 2001 software (POL-LAB, Warsaw, Poland). Reference standards (Merck) were used to identify and quantify sugars by comparison of retention times and UV spectra. Besides the major sugars (sucrose, glucose, and fructose), the samples were screened for minor carbohydrates, including galactose, maltose, melezitose, raffinose, and trehalose. Quantitative analysis of sugars was performed in two samples per sexual phase. From these data, total sugars and the S/[G + F] and G/F ratios were computed. Given the limited number of analytical samples, the nectar sugar composition analysis was conducted to describe phase-related patterns and enable qualitative comparisons rather than to draw population-level conclusions.

### 4.4. Floral Reward—Pollen Production and Pollen Protein

Pollen production was assessed using the method described by Denisow [69]. Anthers were harvested directly prior to dehiscence and placed into pre-weighed glass containers (n = 4 per year). In each container, anthers (n = 100) were randomly collected from flowers (n = 5) originating from different individual plants. The samples were subsequently transferred to an ELCON CL 65 drying oven and maintained at ca. 37 °C for several days to induce anther dehiscence. Pollen was then extracted by adding 3–10 mL of ethanol, which facilitated the release of pollen grains and their separation from anther tissues. The samples were then placed in a drying oven, and after 2–3 weeks, the pollen mass was assessed using an electronic balance (WPS-36; Radwag, Radom, Poland). The pollen production was expressed per flower and per 1 m^2^ (=total pollen production). As the species is multi-staminate, the number of anthers per flower was quantified (n = 10 flowers per year) to enable accurate estimation of pollen mass per flower [70]. In addition, the number of flowers per stem (n = 30 per year) and the number of flowering stems per 1 m^2^ of experimental plots were recorded. These data were used to estimate the total production of pollen and nectar sugars per unit area.

### 4.5. Insect Foragers

Insect foraging activity was monitored during distinct periods of the day (08:00–09:00, 12:00–14:00, and 16:00–17:00) in the area of the experimental plots (n = 4) on three separate days throughout the flowering season. Each observation session lasted 10 min per time period, resulting in a total of 30 min of observation per day. The total number of visitors recorded on each plot per day was summed. The surveys were conducted under typical summer weather conditions favorable for pollinator activity, characterized by clear or partly cloudy skies, calm or light winds, and no precipitation. Visitor abundance was expressed as the average number of individuals per 1 m^2^ over a 1.5 h observation period.

### 4.6. Statistics

Statistical analyses were performed using Statistica 13.1 software (StatSoft, Kraków, Poland). The Mann–Whitney U test, a nonparametric method for independent samples, was employed to compare flowering phenology variables, including flowering onset and duration, between study years. The test was also applied to analyze the differences in the number of flowers between study years. Non-parametric tests were also used to compare insect visitation data.

Analyses of variance (ANOVAs) were employed to examine whether nectar features (nectar mass, nectar sugar concentration, nectar sugar mass), stigma receptivity, and the number of dehiscing anthers differed between consecutive days of anthesis. Data normality was first evaluated using the Shapiro–Wilk test, and log-transformation (Ln) was applied when necessary to meet normality assumptions.

## Figures and Tables

**Table 1:** Flower abundance and nectar production (nectar amount, sugar concentration, and sugar mass per flower) in *Nigella damascena* in 2024–2025.

Year	Number of Flower per Stem	Number of Flower per m^2^ (Thous.)	Mass of Nectar/Flower (mg)	Sugar Concentration (% *w*/*w*)	Mass of Sugars/Flower (mg)
	Mean ± SD	Mean ± SD	Mean ± SD	Mean ± SD	Mean ± SD
2024	7.6 _a_ ± 1.8	0.72 _a_ ± 0.05	0.59 _a_ ± 0.22	45.60 _b_ ± 4.11	0.26 _a_ ± 0.09
2025	10.5 _b_ ± 2.3	1.19 _b_ ± 0.13	0.97 _b_ ± 0.12	39.60 _a_ ± 3.56	0.39 _b_ ± 0.06
mean	9.0 ± 2.5	0.91 ± 0.22	0.78 ± 0.26	42.60 ± 4.82	0.32 ± 0.10

Note: Means followed by the same small letters are significantly different between the years of study at α = 0.05; SD—standard deviation.

**Table 2:** Carbohydrate composition of nectar (% glucose, fructose, and sucrose), sucrose-to-hexose ratio (r), and glucose-to-fructose ratio (rh) during the male and female sexual stages of protandrous *Nigella damascena* flowers. Values are presented as means ± standard deviation (SD).

Sexual Phase	Glucose (G)	Fructose (F)	Sucrose (S)	r = S/(G + F)	rh = G/F
	Mean ± SD	Mean ± SD	Mean ± SD	Mean ± SD	Mean ± SD
Female	17.4 _a_ ± 0.3	11.8 _a_ ± 0.5	70.7 _b_ ± 0.2	2.4 _b_ ± 0.0	1.5 _a_ ± 0.1
Male	36.8 _b_ ± 0.5	14.1 _b_ ± 1.1	49.0 _a_ ± 1.5	1.0 _a_ ± 0.1	2.6 _b_ ± 0.2
Mean	27.1 ± 10.6	13.0 ± 1.5	59.9 ± 11.9	1.7 ± 0.8	2.0 ± 0.6

Note: Different subscript letters indicate significant differences between the sexual phases at *p* < 0.05.

## Data Availability

The raw data supporting the conclusions of this article will be made available by the authors on request.
